# Observations reveal vertical transport induced by submesoscale front

**DOI:** 10.1038/s41598-024-54940-x

**Published:** 2024-02-22

**Authors:** Ruichen Zhu, Haiyuan Yang, Mingkui Li, Zhaohui Chen, Xin Ma, Jinzhuo Cai, Lixin Wu

**Affiliations:** 1 Laoshan Laboratory, Qingdao, China; 2 Present Address: Frontier Science Center for Deep Ocean Multi-Spheres and Earth System (FDOMES) and Physical Oceanography Laboratory, Ocean University of China, 238 Songling Road, Qingdao, 266100 China

**Keywords:** Ocean sciences, Physical oceanography

## Abstract

Submesoscale fronts, with horizontal scale of 0.1–10 km, are key components of climate system by driving intense vertical transports of heat, salt and nutrients in the ocean. However, our knowledge on how large the vertical transport driven by one single submesoscale front can reach remains limited due to the lack of comprehensive field observations. Here, based on high-resolution in situ observations in the Kuroshio-Oyashio Extension region, we detect an exceptionally sharp submesoscale front. The oceanic temperature (salinity) changes sharply from 14 °C (34.55 psu) to 2 °C (32.7 psu) within 2 km across the front from south to north. Analysis reveals intense vertical velocities near the front reaching 170 m day^−1^, along with upward heat transport up to 1.4 × 10^−2^ °C m s^−1^ and salinity transport reaching 4 × 10^−4^ psu m s^−1^. The observed heat transport is much larger than the values reported in previous observations and is three times as that derived from current eddy-rich climate models, whereas the salinity transport enhances the nutrients concentration with prominent implications for marine ecosystem and fishery production. These observations highlight the vertical transport of submesoscale fronts and call for a proper representation of submesoscale processes in the next generation of climate models.

## Introduction

Submesoscale fronts, with horizontal scale of 0.1–10 km and evolution timescale of hours to days, are ubiquitously distributed in the global ocean^1–3^. Generated from several classes of surface frontal instabilities such as frontogenesis, mixed layer instability and turbulent thermal wind^4–12^, they play a vital role in the oceanic energy cascade that acts as a bridge between mesoscale and microscale^13–19^. Compared to the mesoscale fronts with scale from tens to hundreds of kilometers, submesoscale fronts are usually characterized with larger density, temperature, and salinity gradients, which drive stronger secondary circulation in the cross-front and vertical directions^20–23^. Associated with a vertical velocity as large as 10–100 m day^−1^, submesoscale fronts provide efficient routes for the vertical exchange of heat and tracers between the surface and deep ocean^24–27^.

Submesoscale fronts drive an intense net upward heat flux into the surface mixed layer, several times larger than that induced by mesoscale fronts^28–31^, playing an essential role in the heat balance of the ocean surface mixed layer and in air–sea interactions^32–34^. Nutrients fluxes from the ocean interior into the surface euphotic zone are strengthened by submesoscale processes, leading to an enhanced primary production and an increased oceanic carbon uptake; subduction of recently ventilated waters could also be intensified in the presence of submesoscale fronts, affecting the distribution and storage of tracers (e.g., dissolved gases and pollutants) in the global ocean^35–37^. Therefore, a proper estimation of the vertical flux by submesoscale processes is necessary for ocean dynamics and climate research.

However, due to the extremely high resolutions required to resolve submesoscale fronts, direct observations quantifying vertical transports induced by submesoscale fronts remain rare. Some efforts have been made to observe the submesoscale vertical heat flux (VHT). For example, through two nested mooring arrays deployed in the relatively quiescent Northeast Atlantic Ocean, it is found that the submesoscale vertical velocities are most active in winter and spring with a maximum upward heat flux of approximately 50 W m^−2^, larger than that induced by mesoscale^28^. Based on elephant seal and satellite observations, strong upward heat flux driven by submesoscale processes with magnitude of 100 W m^−2^ is reported^29^. These observations have improved our knowledge on submesoscale VHT, but they mainly focus on the time-mean value. To what magnitude of the VHT can reach driven by one submesoscale front remains undiscussed. Though the linkage between submesoscale-induced upward nitrite transport and ecological elements has been observed^38–41^, detailed observations associated with the impact of submesoscale-driven upwelling, such as locations of nutrient rich area and response of marine organism are still missing. Limited by the observation method, these unsolved questions pose hindrances to our understanding of the submesoscale processes and their potential climatic and ecological effect.

**Figure 1:**
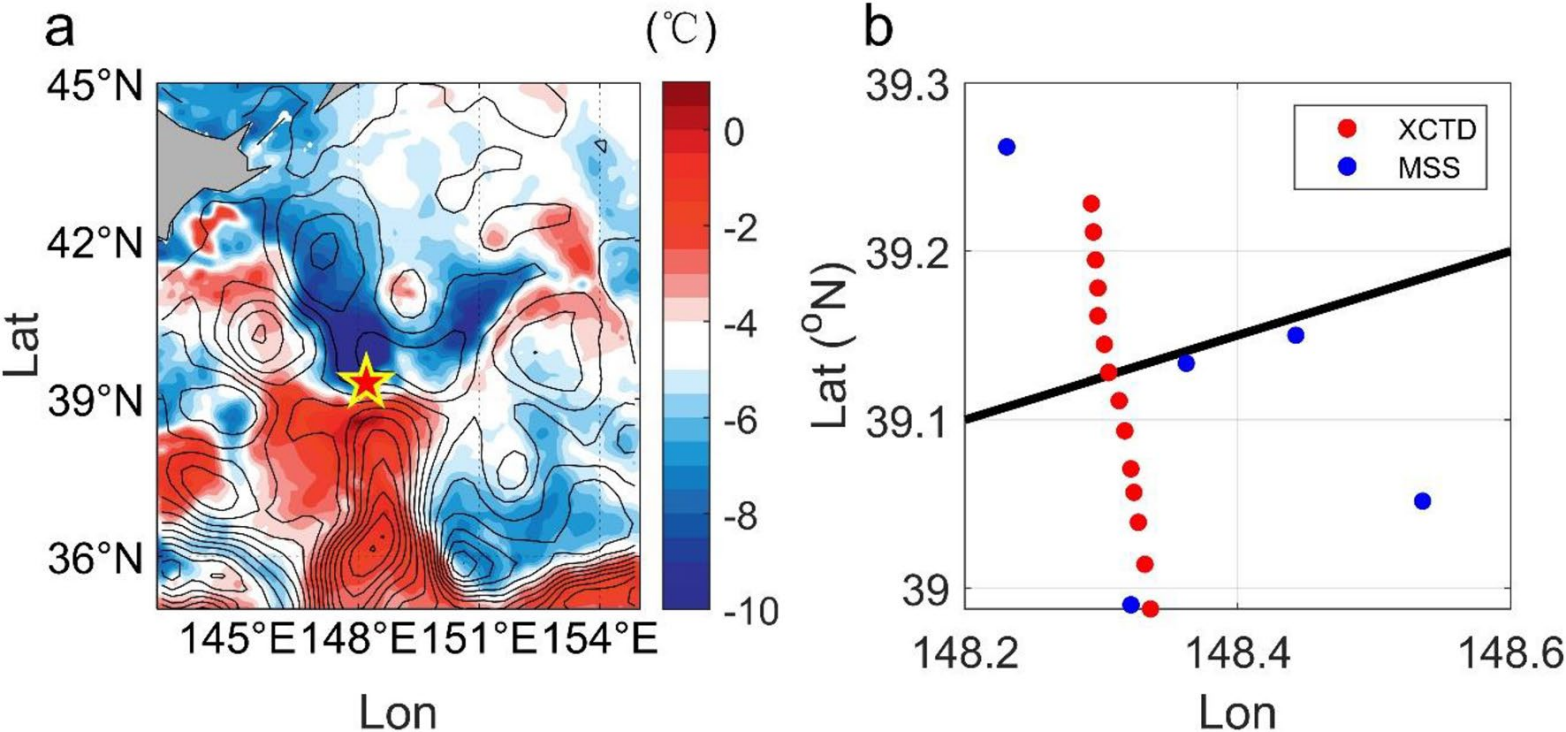
Observations across the submesoscale front. (**a**) SST anomaly (color shadings) and SSH anomaly (black contours) in the KOE region on April 11, 2016. Red pentagram denotes the location of the submesoscale front. (**b**) Position of CTD (red) and MSS (blue) stations. The black line indicates the front. High-resolution hydrographical measurements are conducted in the vicinity of the submesoscale front.

The present study investigates the vertical heat and salinity fluxes of oceanic submesoscale fronts through targeted field observations during the early boreal spring in the Kuroshio–Oyashio Extension (KOE) region (Fig. 1a), a key area abundant with energetic submesoscale fronts^42,43^. Besides, this region is also known to be a highly productive fishing ground, supporting a variety of commercially important marine fishes. The submesoscale front is characterized with large temperature gradient of 6 °C km^−1^ and upward heat transport of 1400 W m^−2^ that is much larger than the values reported in previous observations^29^ and three times as that simulated in mesoscale climate models. The significant vertical fluxes also improve the local ecosystem in the light side of the front within a spatial span of ~ 10 km, by delivering nutrients upward into the euphotic zone. Our results highlight the importance of submesoscale vertical flux and their biogeochemical impacts, and call for a proper representation of these processes in future model simulations.

### Structure and underlying dynamics of the energetic submesoscale front

On April 10, 2016, we carried out a transect across a submesoscale front in the KOE region (Fig. 1a). A satellite-derived sea surface temperature (SST) anomaly image (color shading in Fig. 1a; see "Satellite observations" section in "Method" section) shows that the cold Oyashio water from the subarctic gyre intruded southward to (39° N, 148° E) and encountered a warm-core Eddy shed by the Kuroshio Extension. The intense meridional temperature gradient forms a sharp front with energetic eastward flows as revealed by the dense sea level anomaly (SLA) isolines (contours in Fig. 1a; see "Satellite observations" section in "Method" section). To obtain the structure of front, a high-resolution transection observation in the cross-front direction was conducted along 148° E between 38° 57′ N and 39° 15′ N, using R/V Dongfanghong 2 from 14:21 UTC on April 10 to 12:45 UTC on April 11, 2016 (Fig. 1b). The horizontal velocity fields are collected by a shipboard Acoustic Doppler Current Profiles (ADCP) with an averaged sampling time of 1 min and a bin size of 16 m in the upper 800 m. Fourteen Expendable Conductivity/Temperature/Depth (XCTD) profilers are deployed every ~ 2 km to observe the upper-1000 m ocean (red dots in Fig. 1b). The obtained temperature/salinity (T/S) data are interpolated onto 2-km horizontal and 1-m vertical grids before using. In the vicinity of the front, observation based on a microstructure system (MSS) is conducted to measure the turbulence shear (blue dots in Fig. 1b). Then the turbulent kinetic energy dissipation rate (ε) is calculated by fitting the observed shear spectrum to the Nasmyth spectrum^44^. During the shipboard observation period, the front is approximately steady without substantial changes (see Supplementary Fig. S1), which serves as an ideal opportunity for investigating its characteristics and underlying dynamics.

**Figure 2:**
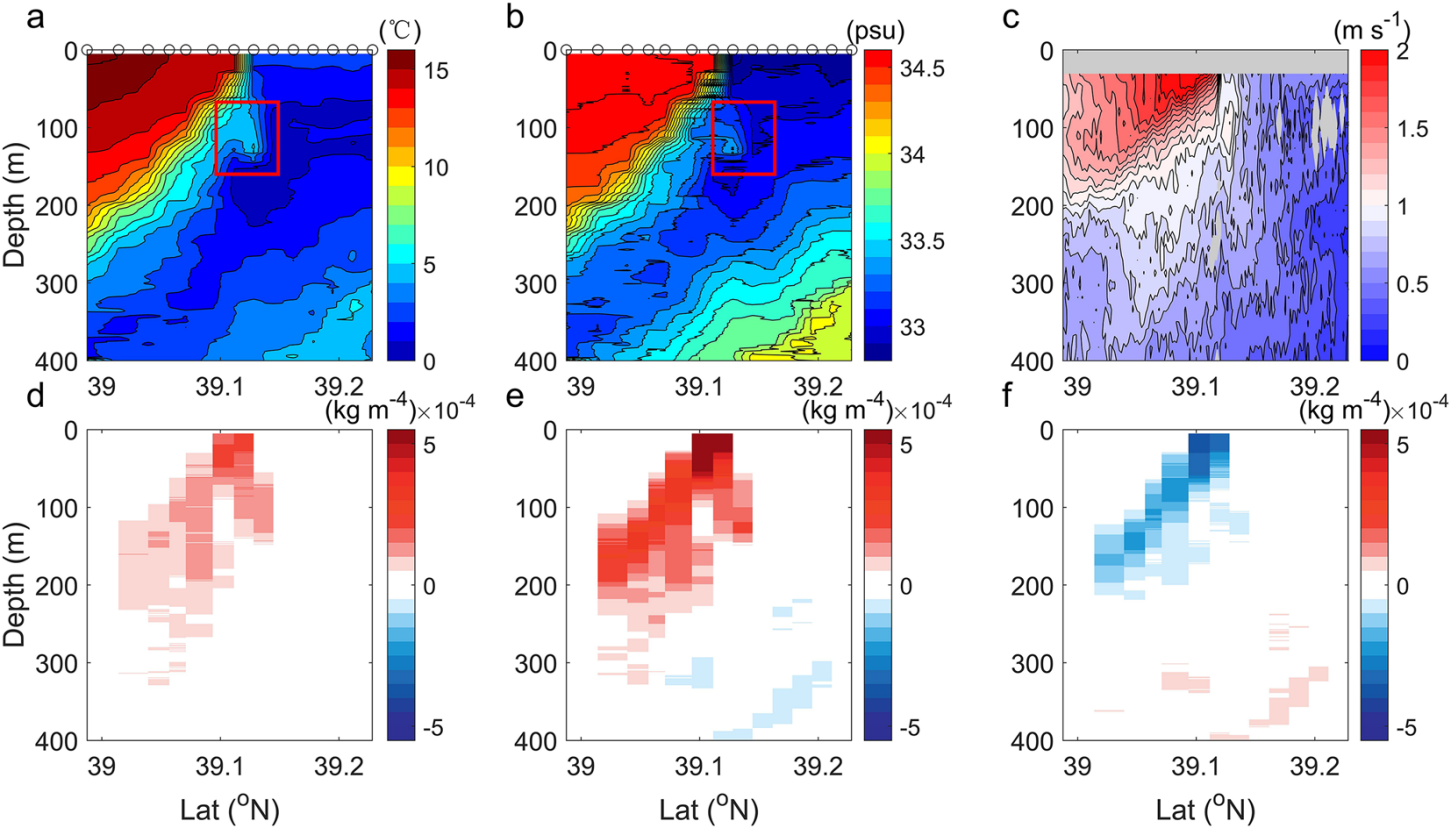
Characteristics of the submesoscale front. (**a**) Temperature, (**b**) salinity, (**c**) zonal velocity and d meridional gradient of density (*ρ*_*y*_). e Thermal (*ρ*_*y*_^*T*^) and f haline (*ρ*_*y*_^*S*^) contributions to *ρ*_*y*_ in (**d**). Red boxes in (**a**) and (**b**) denote the depression structure of isopycnals. Observations reveal an intense submesoscale front with large velocity and cross-front changes.

The submesoscale front shows large cross-front variability (Fig. 2). Across the front from 39.0° to 39.1° N, a rapid drop in oceanic temperature in the upper 50 m (from 14 to 2 °C) within 2 km (Fig. 2a) is detected with temperature gradient reaching ~ 6 °C km^−1^. Similarly, the upper-50 m salinity field also presents a gradient of ~ 0.96 psu km^−1^ (Fig. 2b). Both the temperature and salinity gradients decrease with depth. Accompanied by the strong temperature and salinity gradients, velocity core with magnitude exceeding 2 m s^−1^ between 30 and 60 m depth on the light side of the front is captured by ADCP (Fig. 2c). In the vertical direction, the front extends to 250 m with an isopycnal slope of 0.017 (see "Isopycnal slope" section in "Method" section; Fig. 2a and b). The large cross-front change of temperature and salinity induces a density gradient (Fig. 2d; see "Density gradient" section in "Method" section) with maximum of 2.2 × 10^−4^ kg m^−4^, equivalent to a buoyancy gradient of 2.15 × 10^−6^ s^−2^, which is two (four) times as that of observed submesoscale fronts in the Gulf Stream (Southern Ocean)^5,29^ and comparable to the value induced by the Kuroshio Extension main stream^13^. By decomposing the density into temperature-induced and salinity-induced components, it is found that the density gradient is dominated by cross-front temperature change and partly compensated by salinity variability (Fig. 2e,f), consistent with previous estimations^45,46^.

**Figure 3:**
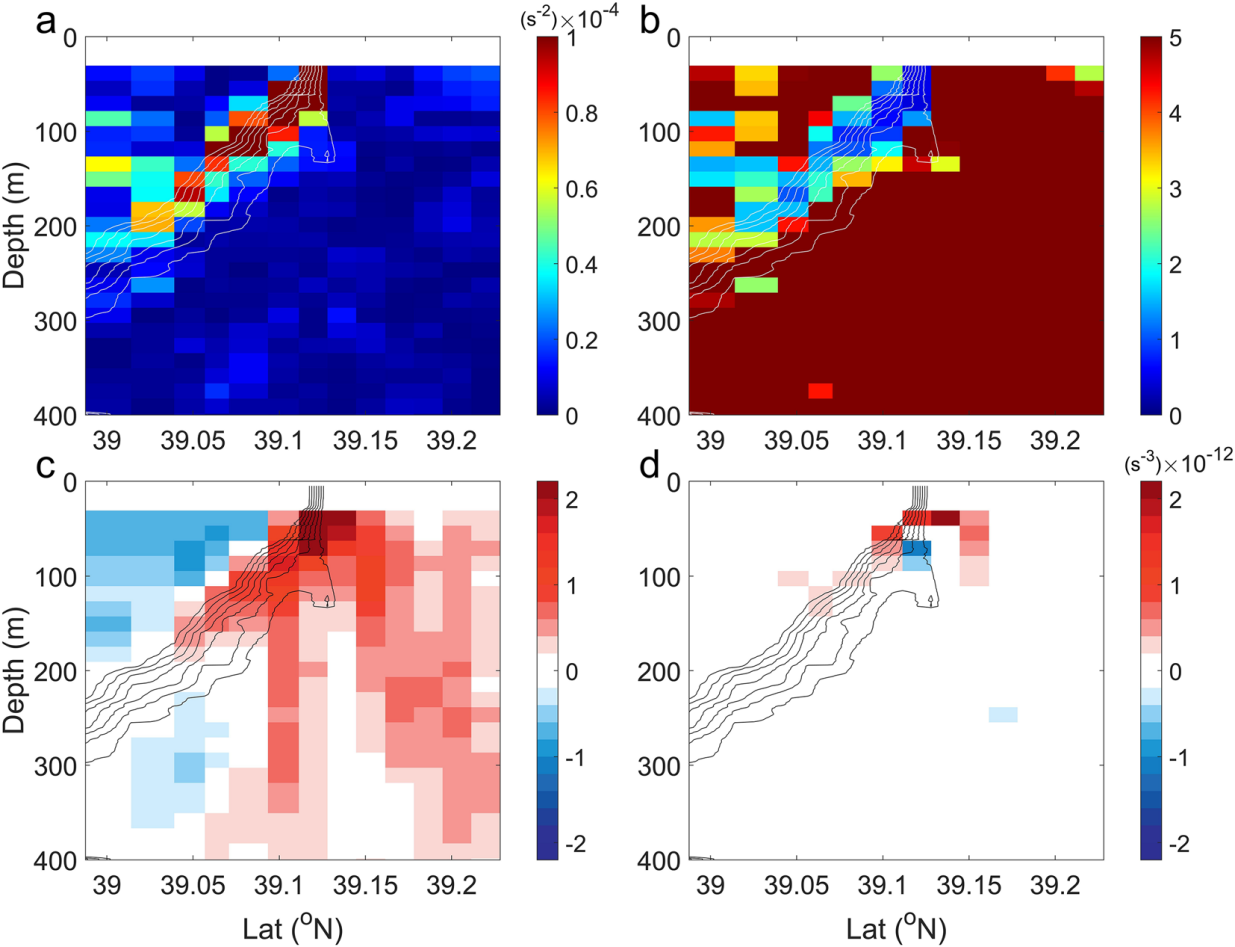
Dynamics of the submesoscale front. Vertical section of (**a**) the velocity shear variance (*S*^2^), (**b**) Richardson number (Ri), (**c**) Rossby number (Ro) and (**d**) horizontal advection term of frontogenesis function (F_ADV_). Black contours are in-situ temperature with an interval of 2 °C. Frontogenesis accounts for the generation of the intense submesoscale front.

The intense cross-front changes favor the development of oceanic instability^2,3^. Along the front interface, velocity shear variance (*S*^2^ =|∂*u*/∂*z*|^2^ +|∂*v*/∂*z*|^2^, where *u* and *v* are zonal and meridional velocity measured by ADCP) is very large in the upper 100-m depth, reaching a maximum value of 3 × 10^−4^ m^2^ s^−4^ near the surface (Fig. 3a). The intense vertical relative vorticity (*ζ*≈ − ∂*u*/∂*y*) and vertical shear lead to Rossby number (Ro =|*ζ|*/*f*) and Richardson number (Ri = *N*^2^/*S*^2^) of *O* (1) (Fig. 3b and c; *f* is the Coriolis parameter and *N*^2^ represents the Brunt–Väissälä frequency). These two non-dimensional numbers suggest that the front is in an ageostrophic regime and subjects to energetic submesoscale variability that can effectively draw kinetic energy from background flows and drive a forward energy cascade towards turbulent dissipation^47^. Within the narrow zone where Ri < 1, the *ε* derived from the MSS measurements is five times larger than an estimation for energy input to the near-inertial waves from the wind (see "Slab model" section in "Method" section), and is an order of magnitude greater than that at other stations away from the front. The Ertel potential vorticity is positive over the entire frontal region (see Supplementary Fig. S2) and the mixed-layer deformation radius of *O*(1 km) is less than the length scale of front (see “Ertel PV” section and "Mixed-layer deformation radius" sections in "Method" section), implying that the centrifugal-symmetrical instability and mixed-layer instability contribute less to the turbulent dissipation^48,49^. Therefore, it is possible that the shear instability provides the main energy source for the enhanced turbulent mixing.

To investigate the underlying dynamic processes governing the frontal evolution, we calculate the horizontal advection term (F_ADV_; see “Frontogenesis function” section in “Method” section) in the frontal tendency equation, which describes the contribution of frontogenesis mechanism in the development of submesoscale front. As shown in Fig. 3d, F_ADV_ is positive along the front interface with maximum of 2 × 10^−12^ s^−3^ at 30 m and its magnitude decays quickly away from the front, demonstrating that the contribution of advective frontal tendency term is frontogenetic, corresponding to the intensification of velocity gradients at the front^50^.

### Intense vertical transports and substantial biochemical effects

Based on previous studies^20,51^, the frontogenesis processes are not only characterized by the horizontal gradient intensification with large lateral strain and convergence, but also accompanied by intense vertical motions. The finite Ro and small Ri lead to the loss of geostrophic balance and then an across-front ageostrophic secondary circulation^2,52,53^, which tends to relax the tilted thermocline and restore the geostrophic and thermal wind balance. As the frontal jet accelerates, anticyclonic (cyclonic) vorticity keeps increasing on the light (heavy) side of the front, which requires horizontal flows to diverge (converge) for the conservation of potential vorticity. As such, vertical motions are induced. Although the vertical velocity is hard to be measured directly, an abrupt depression of isopycnals can be clearly seen on the heavy side beneath the front interface (red box in Fig. 1a and b), indicating the presence of the downwelling cell^25^. In the next, we will diagnose the *w* based on the dynamical-based framework.

**Figure 4:**
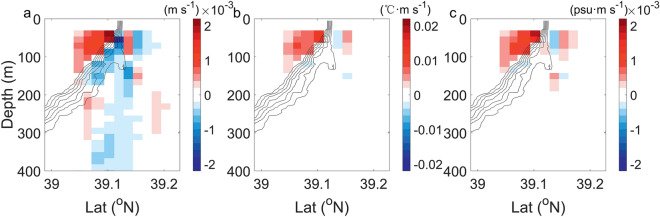
Vertical velocity and transports induced by the submesoscale front. (**a**) Vertical velocity (*w*), (**b**) VHT and (**c**) VST. Black contours are in-situ temperature with an interval of 2 °C. The submesoscale front is characterized by strong vertical velocity and transports.

By solving the 2-dimensional generalized *ω*-equation using the observed *T*/*S*, *ε* and horizontal velocity, the diagnosed *w* field is obtained (Fig. 4a; see “Estimation of ocean vertical velocity” section in “Method” section). As the theory predicted, *w* is downward (upward) on the heavy (light) side of the front, with a vertical extent up to 150 m depth (Fig. 4a). It reaches its maximum at the location of the maximum temperature gradient at ~ 50 m depth, and the magnitudes of maximum upward and downward velocities are almost same, about 170 m d^−1^, which is larger than most of the vertical velocity observed in the vicinity of submesoscale fronts^54,55^. The intense vertical motions enhance the exchange of heat, salt and material between the upper layer and ocean interior. In our observation, the frontogenesis-induced VHT (see ‘VHT and VST’ in “Method” section) is positive above 150 m depth (Fig. 4b), which is indicative of the net upward heat across the front. On the light side of the front, VHT reaches its maximum values of 1.4 × 10^−2^ °C m s^−1^, coincident with the location of the maximum upward *w*.

**Figure 5:**
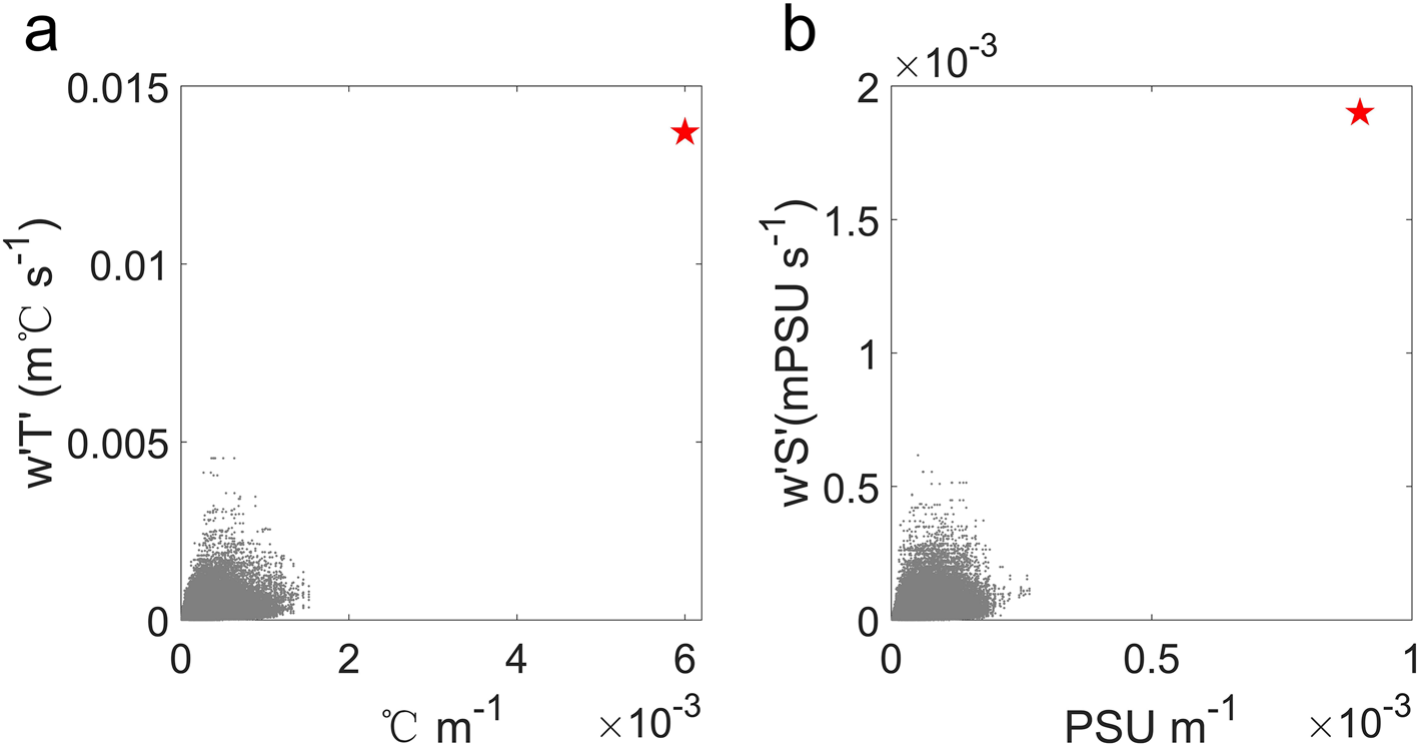
Comparison between the modeled and observed VHS and VST. (**a**) A scatter plot of the |∇*T*| and VHT derived from the CESM product (grey dots) and from observation (red pentagram). (**b**) is same as (**a**) but for the |∇*S*| and VST. The climate model-simulated VHT and VST are much weaker than the observed value.

Our observation represents the first observational evidence of such strong VHT for a single submesoscale front. Although a recent observation by an elephant seal in the Antarctic Circumpolar Current region shows that the deep submesoscale fronts can induce an anomalous upward heat transport comparable to this study^29^, their definition of anomalous transport is based on the time-mean over the entire observation period, which may potentially overestimate the VHT. Besides missing in previous observation, the strong VHT revealed in our observation is neither captured by widely-used mesoscale-resolving climate models. Here, we use the 4-year output from the CESM products (see ‘CESM model simulation’ in "Method" section) with a 1/10° horizontal resolution to calculate the maximum VHT within the upper 150-m layers as a function of temperature gradient |∇*T*| at each grid from 36° to 42° N and 145°–150°E. As shown in Fig. 5a, both the front intensification and upward heat transport simulated in CESM model are much weaker than the observed values. In particular, model-simulated VHT is approximately 30% of observed value. This suggests that the climate models are very likely to underestimate the VHT caused by the submesoscale dynamics, which may further result in a bias of the air–sea heat exchange^32^.

**Figure 6:**
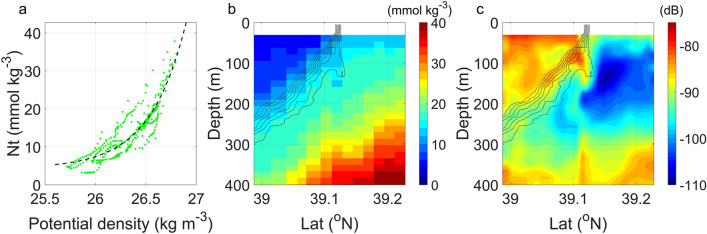
The biochemical impact of the submesoscale front. (**a**) Nitrate–density relationship from the WOA2018 data. Vertical section of (**b**) nitrate concentration (*N*_*t*_) and (**c**) estimated backscattering coefficient (*S*_*v*_). Black contours are in-situ temperature with an interval of 2 °C. Upward nutrient delivery drives the growth of biomass.

In addition to VHT, the submesoscale front drives an intense vertical salinity transport (VST; see “VHT and VST” section in “Method” section) as well. Similar to VHT, the observed VST can reach up to 1.9 × 10^−3^ psu·m s^−1^ (Fig. 4c), which is also about 3 times as the simulated magnitude in the CESM model (Fig. 5b). Such strong VST indicates effective upward nutrient transport. Considering that there exists a good relationship between the nutrient content and potential density^56,57^, we perform a least square fit to the *N*_*t* _− *ρ* scatter using WOA2018 (see “WOA2018” section in “Method” section) April climatological T/S and nitrate (*N*_*t*_) profiles. As shown in Fig. 6a, their relationship can be well described by the exponential function:

1 $$N_{t} = {1}{\text{.021}} \times {\text{e}}^{{{2}{\text{.791}}\rho }} { + 4}{\text{.287, 25}}{.6 < }\rho < 26.9,$$

where the nutrients are treated as passive tracer in the vicinity of front. Then, plugging the observed *ρ* profiles into Eq. (1) yields the cross-front *N*_*t*_ field in Fig. 6b. Despite high nutrients from Oyashio water being distributed on the heavy side of the front, *N*_*t*_ increases with depth on both sides of the front. Thus, the upwellings on the light side will lead to the upward transport of eutrophic waters from ocean interior to surface layers, favoring the growth of biomass. The vertical velocities can be further decomposed into the movement of isopycnal surface and the along-isopycnal motion (*w*_*uplift*_ and *w*_*iso*_; see “Vertical velocity decomposition” section in "Method" section; see Supplementary Fig. S3), both of which exhibit comparable magnitudes and nutrient transport capabilities. Figure 6c reveals that the backscattering coefficient (*S*_*v*_; see “Backscattering coefficient” section in “Method” section) has a similar pattern to the T/S profiles. Surprisingly, the same depression structure of isopycnals is also detected in the *S*_*v*_ field where the high *S*_*v*_ on the light side of the front dips beneath the front interface, again demonstrating the presence of strong downwelling on the heavy side of the front. High *S*_*v*_ values are distributed on the light side of the front, ~ 20 dB larger than that on the heavy side, corresponding to the high biomass in the upwelling region. The convergence of biomass near the front favors the increase of predators, which is important for the maintenance of marine ecosystem and fishery production.

### Summary and implications

In this study, we investigate an unexpectedly intense submesoscale front observed in the Kuroshio–Oyashio Extension region using high-resolution in situ measurements in April 2016. Generated by active frontogenesis, the front is characterized by diagnosed vertical velocities reaching 170 m day^−1^. It drives formidable vertical fluxes along the front, with an upward heat flux of up to 1.4 × 10^−2^ °C m s^−1^, which is much larger than the values reported in previous observations and is three times larger as that derived from current climate models. Besides, we find that the submesoscale front transports nutrients in the vertical direction that is also three times larger as that of climate models, with significant implications for marine ecosystem and fishery production. Therefore, climate models with higher resolutions and more accurate submesoscale parameterizations are needed to better simulate the submesoscale fronts and associated climate and ecological effects.

## Method

### Satellite observations

The sea surface temperature (SST) data is obtained from The Operational Sea Surface Temperature and Ice Analysis (OSTIA) system. This product has a horizontal resolution of 0.05°, using in-situ and satellite data from both infrared and microwave radiometers. SST anomaly is calculated by subtracting the long-term mean value from the raw data. The 1/4° daily sea level anomaly (SLA) data derived from Copernicus Marine Environment Monitoring Service (CMEMS) are applied to describe the surface information of the front.

### Isopycnal slope

The slope of the front was estimated by taking the ratio of the vertical distance between isopycnals to the horizontal distance between closely spaced XCTD stations.

### Density gradient

The density (*ρ*) and buoyancy (*b* = *−* *gρ/ρ*_0_) of seawater are calculated from the observed temperature and salinity, where *g* is the gravitational acceleration and *ρ*_0_ the reference density set as 1025 km m^−3^. The density profile is estimated using the simple equation of state:

$$\rho = \rho_{0} [1 - \alpha (T - T_{0} ) + \beta (S - S_{0} )],$$

where *α* = 1.7 × 10^−4^ K^−1^ and *β* = 7.6 × 10^−4^ psu^−1^ are the coefficient of thermal expansion and saline contraction, *T*_0_ = 10 °C and *S*_0_ = 35 psu are the reference values of *T* and *S*, respectively. The thermal (*ρ*_*y*_^*T*^) and haline (*ρ*_*y*_^*S*^) contributions to the meridional density gradient are defined as:

$$\rho_{y}^{T} = - \rho_{0} \alpha \frac{\partial T}{{\partial y}},\rho_{y}^{S} = \rho_{0} \beta \frac{\partial S}{{\partial y}}.$$

### Ertel PV

The potential vorticity is defined as the dot product of the absolute vorticity ***ω*** = *fz* + ∇ × **u** and the buoyancy gradient ∇*b*, where *f* is the Coriolis parameter **z** is the unit vertical vector, *g* is the gravitational acceleration, and *σ* is the potential density referenced to the surface.

### Mixed-layer deformation radius

The mixed-layer deformation radius can be expressed as *NH*/*f*, where *H* is the mixed layer depth that is defined as the depth below the sea surface where the density increases by 0.03 kg m^−3^ compared to the surface density.

### Slab model

A simple slab model^58^ is conducted by inputting the hourly-averaged surface wind derived by the vessel monitoring system (VMS) on April 10–11, 2016 to indirectly assess the potential influence of inertial waves on the vertical motions at the front.

### Frontogenesis function

Following the previous studies^7,59^, F_ADV_ is defined based on velocity gradient instead of buoyancy gradient because the frontogenesis evolution could be more correctly captured by the change of velocity gradient:

$${\text{F}}_{ADV} = - \left( {\frac{\partial u}{{\partial x}}\nabla_{h} u + \frac{\partial u}{{\partial y}}\nabla_{h} v} \right) \cdot \nabla_{h} u - \left( {\frac{\partial v}{{\partial x}}\nabla_{h} u + \frac{\partial v}{{\partial y}}\nabla_{h} v} \right) \cdot \nabla_{h} v \approx - \frac{{\partial^{2} u}}{{\partial y^{2} }}\frac{\partial v}{{\partial y}} - \frac{{\partial^{3} v}}{{\partial y^{3} }},$$

where ∇_*h*_ is the horizontal gradient operator.

### Estimation of ocean vertical velocity

The generalized Q-vector *ω*-equation^60^ is applied here to diagnose the vertical velocity in the upper ocean. In comparison to the Quasi-Geostrophic (QG) version of *ω*-equation, this approach introduces the ageostrophic sources of vertical velocity, which arises from the retroaction effect of ageostrophic flows on itself. Therefore, the generalized *ω*-equation is a powerful tool for the evaluation of the vertical motions in submesoscale dynamic processes (e.g., fronts, filaments and eddies). The generalized *ω*-equation can be written as follows:

$$\begin{gathered} f^{2} \frac{{\partial^{2} w}}{{\partial z^{2} }} + \nabla_{h} (N^{2} \cdot \nabla_{h} w) = \nabla_{h} \cdot \{ \underbrace {{ - 2(\nabla_{h} {\mathbf{u}}_{g} )^{{\text{T}}} \cdot \nabla_{h} b}}_{{Q_{tg} }}\underbrace {{ - 2(\nabla_{h} {\mathbf{u}}_{a} )^{{\text{T}}} \cdot \nabla_{h} b}}_{{Q_{tag} }}\underbrace {{ - f[\nabla_{h} ({\mathbf{k}} \times {\mathbf{u}})]^{{\text{T}}} \cdot \frac{{\partial {\mathbf{u}}_{a} }}{\partial z}}}_{{Q_{dag} }}\underbrace {{ - f{\mathbf{k}} \times \frac{D}{Dt}(\frac{{\partial {\mathbf{u}}_{a} }}{\partial z})}}_{{Q_{dr} }} \hfill \\ \underbrace {{ - f\frac{\partial }{\partial z}[{\mathbf{k}} \times D_{H} ({\mathbf{u}})] - f\frac{\partial }{\partial z}[{\mathbf{k}} \times D_{V} ({\mathbf{u}})]}}_{{Q_{dm} }} + \underbrace {{\nabla_{h} [D_{V} (b)]}}_{{Q_{th} }}\} , \hfill \\ \end{gathered}$$

where *w* the vertical velocity, *N* the buoyancy frequency, ∇_*h*_ the horizontal gradient operator, **u** = **u**_*g*_ + **u**_*a*_ the horizontal velocity that can be further decomposed into geostrophic and ageostrophic component, *D*_*H*_ and *D*_*V*_ the horizontal and vertical mixing, respectively. Physically, the first two terms on the right-hand size are the kinetic deformation caused by the geostrophic and ageostrophic horizontal flow (*Q*_*tg*_ and *Q*_*tag*_). The third and fourth terms denote the forcing by the thermal wind imbalance and its material derivative (*Q*_*dag*_ and *Q*_*dr*_). And the last two terms represent the momentum and buoyancy turbulent forcings (*Q*_*dm*_ and *Q*_*th*_). Based on a MITgcm simulation with 1/48° horizontal resolution, it is found that the *Q*_*dag*_ and *Q*_*dr*_ contribute less to the *w* magnitude than other forcings^61^. Combined with the transection data of observed front, we only take *Q*_*tg*_, *Q*_*tag*_ and *Q*_*dm*_ into account and simplify the Eq. (1) into a 2-dimensional plain (y, z) under an assumption that the along-front variation is smaller than the cross-front variation:

$$f^{2} \frac{{\partial^{2} w}}{{\partial z^{2} }} + \frac{\partial }{\partial y}\left( {N^{2} \frac{\partial w}{{\partial y}}} \right) = - 2\frac{\partial }{\partial y}\left( {\frac{\partial v}{{\partial y}}\frac{\partial b}{{\partial y}}} \right) + f\frac{\partial }{\partial y}\frac{{\partial^{2} }}{{\partial z^{2} }}\left( {\kappa \frac{\partial u}{{\partial z}}} \right),$$

where the *κ* = *Γε*/*N*^2^ is the vertical diffusion coefficient with a constant *Γ* of 0.2^62^. Before the *w* inversion, all velocity and *ε* profiles are linear interpolated onto the XCTD sites.

### VHT and VST

The VHT and VST are defined as *wT'* and *wS'*, where *w* is the vertical velocity, *T'* and *S'* the temperature and salinity anomaly to the mean value of all profiles, respectively.

### CESM model simulation

To compare the magnitude differences of ocean vertical heat transport between the in-situ observations and numerical simulations, an Eddy-resolving global coupled climate model, Community Earth System Model (CESM), is used in this study. The model is configured and run at a horizontal resolution of 0.25° Community Atmospheric Model version 5 (CAM5) for the atmosphere components and 0.1° Parallel Ocean Program version 2 (POP2) for the ocean components. The ocean and atmosphere models interact with each other every 6 h, during which POP2 provides sea surface temperature and velocity to CAM5, then CAM5 updates downward fluxes and feedback to POP2 using the surface layer scheme. The K-profile parameterization (KPP) turbulent mixing closure scheme is used for vertical mixing. The ocean component of the CESM is initialized with a static ocean with potential temperature and salinity of mean January climatology from World Ocean Atlas (WOA). Other components are started from restart files of previous simulations. The radiation forcing is set to preindustrial (1850) conditions and kept constant for 250 years. After the 250-year spin-up, the historical forcing from 1850 to 2005 is applied to the model, during which the model provides monthly three-dimensional output. In particular, daily averaged ocean variables during 2000–2003 are saved for use in this study. It is worth mentioning that the temperature anomalies used to calculate VHT in this model are obtained by subtracting the area mean of a 0.2° × 0.2° box.

### World Ocean Atlas 2018 (WOA18) product

In order to access the distribution of nutrient concentrate across the submesoscale front, the April climatological profiles (from 2005 to 2017) of temperature, salinity and nutrients are derived from the WOA2018 product to fit the relationship between the nutrients and density. Here data in the upper 250 m within 146–150° E and 36°–42° E are used.

### Vertical velocity decomposition

The vertical motion along isopycnal surfaces (*w*_*iso*_) and movement of isopycnal surfaces (*w*_*uplift*_) can be expressed as follows^63^:

$$\left\{\begin{array}{c}{w}_{iso}={\mathbf{u}}_{{\text{div}}}\cdot \nabla h+\frac{\left|\nabla h\right|}{1+{\left|\nabla h\right|}^{2}}\left(\frac{\partial h}{\partial t}+{\mathbf{u}}_{{\text{rot}}}\cdot \nabla h\right)\approx {v}_{div}\frac{\partial h}{\partial y}\\ {w}_{uplift}=w-{w}_{iso}\end{array},\right.$$

where **u**_**div**_ and **u**_**rot**_ are divergent and rotational component of the total velocity, and *h*(*x*,*y*) the height of isopycnal surface.

### Backscattering coefficient

Based on the sonar equation, *S*_*v*_ can be estimated from ADCP echo amplitude records for characterizing the zooplankton population in the water column^64,65^, which can be written as:

$$S_{V} = C + 10\log_{10} [(T_{x} + 273.16)R^{2} ] - 10\log_{10} (L) - 10\log_{10} (P) + 2\alpha R + K_{c} (E - E_{r} ),$$

where *C* is the parameter of instrument set as − 163.3 dB, *T*_*x*_ transducer temperature (°C), *R* the distance along the beam to the scatterer, *L* transmit pulse length, *P* transmit power, *α* the absorption coefficient of water, *K*_*c*_ the scale factor set as 0.5, *E* echo intensity and *E*_*r*_ the reference level of *E* for each beam.

### Supplementary Information

Supplementary Figures.

## Data Availability

All observational data is uploaded to the Open Science Framework repository (10.17605/OSF.IO/YMDZF). Both SSTA and SLA data are obtained from Copernicus Marine Environment Monitoring Service at 10.48670/moi-00165 and 10.48670/moi-00148, respectively, after registering at https://resources.marine.copernicus.eu/registration-form. The WOA18 product is also provided by National Centers for Environmental Information at https://www.ncei.noaa.gov/data/oceans/woa/WOA18/DATA/. The CESM model source code and associated instructions are provided by Laoshan Laboratory and can be downloaded from https://github.com/ihesp/CESM_SW.
