# Supplementary material for: Observations reveal vertical transport induced by submesoscale front
Source: Sci Rep. 2024 Feb 22;14:4407. doi: 10.1038/s41598-024-54940-x (PMC10883923; doi:10.1038/s41598-024-54940-x)
Supplement: Supplementary file 1 — Supplementary Figures. [file 41598_2024_54940_MOESM1_ESM.docx]

**Supplementary Information *for***

**Observations reveal vertical transport induced by submesoscale front**


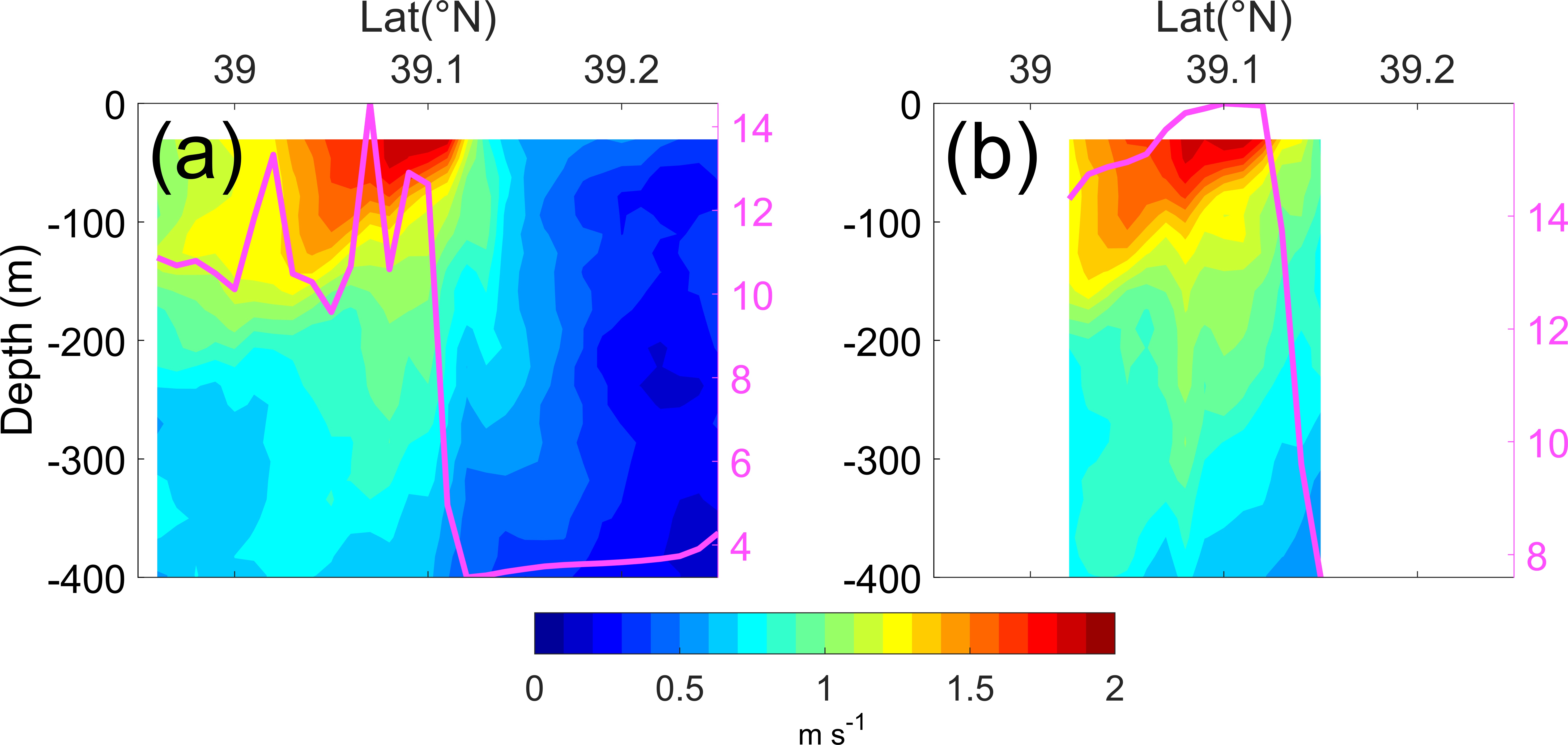


**Figure R1.** Zonal velocity on April 10, 2016 at (a) 15:00 UTC and (b) 23:00 UTC. The magenta lines denote shipboard SST records across the front. During the shipboard observation period, the front is approximately steady without substantial changes.


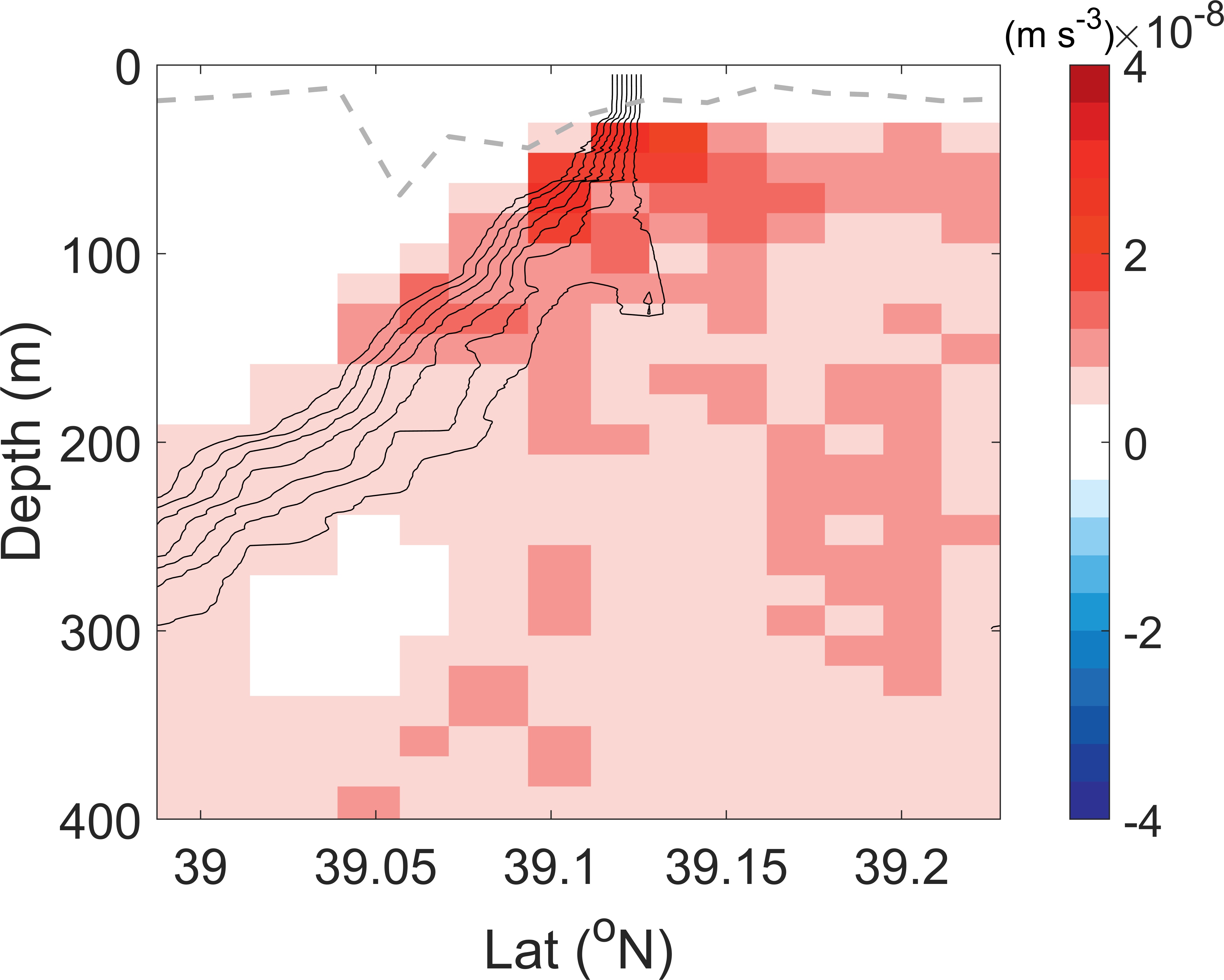


**Figure R2.** Potential vorticity field. Black contours are in-situ temperature with an interval of 2 ℃. Dashed line denotes the mixed layer depth.

S
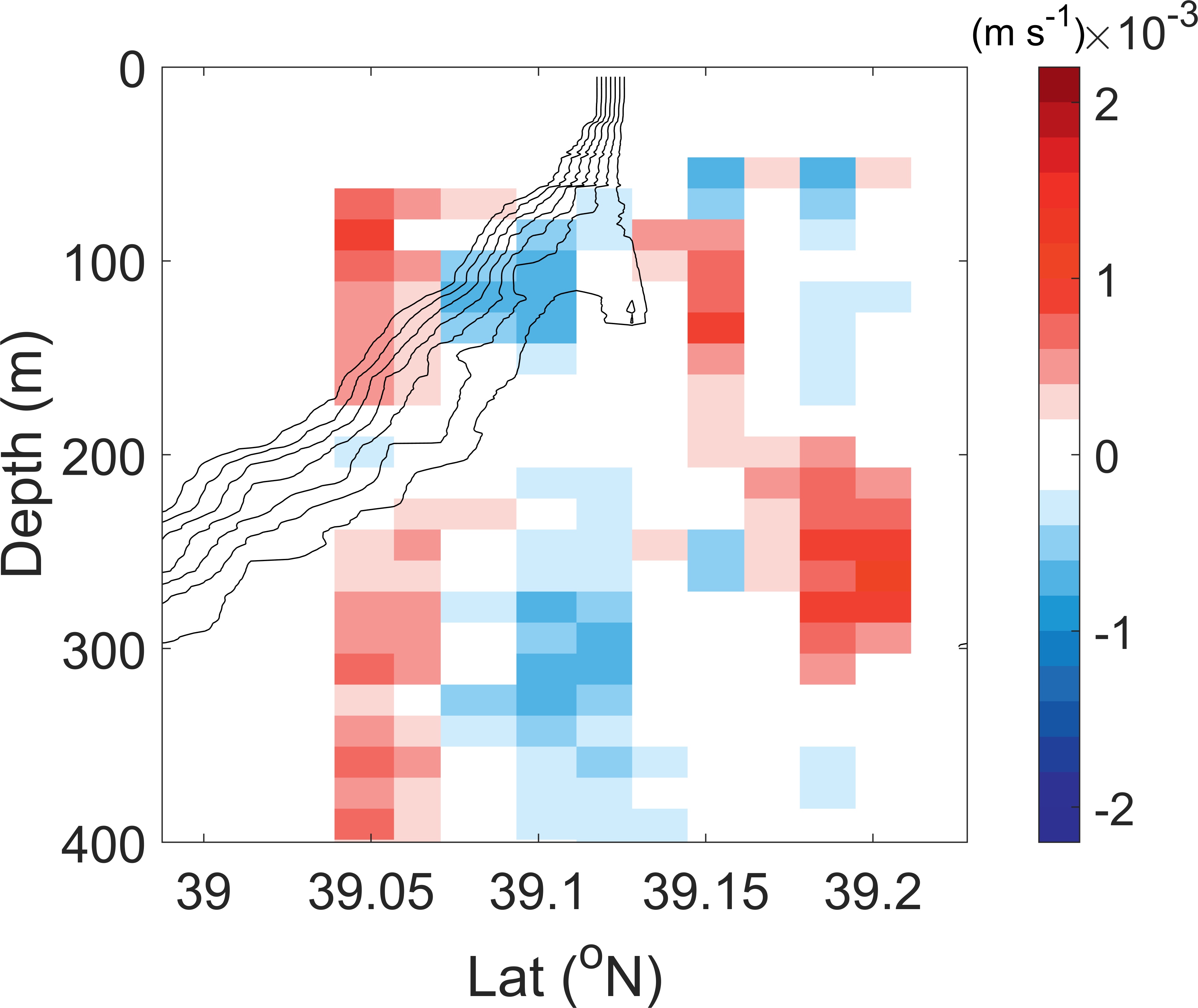


**Figure R3.** Vertical motion along isopycnal surfaces. Black contours are in-situ temperature with an interval of 2 ℃.
